# Microbial Landscape of Bull (*Bos taurus*) Ejaculate: Assessment of Diversity and Link to Fertility

**DOI:** 10.3390/ani16081257

**Published:** 2026-04-20

**Authors:** Elena A. Yildirim, Evgeniy Finageev, Kseniya Sokolova, Nataliia Patiukova, Maria Shubina, Angelina Belikova, Elena Korochkina

**Affiliations:** 1 Molecular Genetics and Microbiomics Laboratory, BIOTROF+ Ltd., Pushkin, St. Petersburg 196602, Russia; deniz@biotrof.ru (E.A.Y.);; 2 Federal State Budgetary Educational Institution of Higher Education, St. Petersburg State Agrarian University, Pushkin, St. Petersburg 196605, Russia; 3 Federal State Budgetary Educational Institution of Higher Education, St. Petersburg State University of Veterinary Medicine, St. Petersburg 196084, Russiamaris.shubi@yandex.ru (M.S.);

**Keywords:** bulls, semen quality, microbiota, NGS, fertility, microbial landscape

## Abstract

The composition of the sperm microbiome in bulls (*Bos taurus*) may influence reproductive function. This study aimed to examine the taxonomic composition of the sperm microbiome in bulls to establish its possible link with semen quality. The study investigated the composition of the sperm microbiome of 21 Holstein bulls with varying sperm quality, including subfertile animals. The quality of sperm was assessed using standard methods. Analysis of the microbial community of the semen was performed using targeted next-generation sequencing (NGS). The data indicated significant species richness of microorganisms in the semen of the studied bulls. At least 15 different bacterial phyla were identified in the semen samples. Significant differences in the microbiome composition were revealed in samples of different sperm quality groups. Higher relative abundance of Actinobacteriota (from 1.9 ± 0.12% to 14.1 ± 1.02%) was observed in bulls with excellent semen quality. *Bacteroides fragilis* was detected in 75% of samples of low-quality sperm. At the same time, the analysis revealed individual differences in the microbial composition of samples. Our results indicate a link between the microbiome composition and qualitative indicators of the sperm of bulls. This suggests that the identified taxonomic markers may serve as additional bioindicators for assessing fertility in males.

## Simple Summary

This study investigated whether the microscopic organisms living in bull semen, known as the microbiome, could affect fertility. We analyzed semen samples from 21 Holstein bulls, comparing the types and amounts of bacteria present with the quality of the sperm. In the samples, we discovered a diverse community of over 15 different major bacterial groups. Bulls with excellent semen quality had a higher proportion of a bacterial group called Actinobacteriota. Furthermore, a specific bacterium, *Bacteroides fragilis*, was found in three-quarters of the low-quality samples. These findings suggest that the makeup of the semen microbiome is connected to male fertility in bulls. This is valuable because it means that in the future, farmers and veterinarians might be able to use the presence of these helpful bacteria as a new tool, or bioindicator, to quickly and accurately assess a bull’s breeding potential, leading to more efficient livestock management.

## 1. Introduction

The fertility of sires plays an important role in the efficiency of dairy and beef cattle breeding [1]. Maintaining high and stable reproductive performance in males remains a serious challenge, as a significant proportion of cases of non-pregnancy during artificial insemination are caused by bull subfertility because of low sperm quality [2]. Under conditions of intensive livestock farming and common use of assisted reproductive technologies such as artificial insemination, the sperm quality of high-value sires directly determines the genetic progress and profitability of entire herds. Despite advances in genetics and veterinary medicine, a significant proportion of subfertility cases still cannot be explained from the point of classical physiology [3].

Traditionally, the evaluation of reproductive potential (Breeding Soundness Examination, BSE) is used as the main tool for analyzing bull fertility [4]. Sperm quality is assessed within the BSE using standard markers of sperm viability, including morphology, motility, concentration, etc. [5]. However, the fact that even sperm samples with normal parameters sometimes show low fertilizing ability indicates the need to search for additional fertility markers. Furthermore, the BSE protocol evaluates the phenotypic outcome but does not identify the etiology of fertility problems [4].

In recent years, increasing attention has been paid to the role of the microbiome in various aspects of animal productivity and health status, including reproductive function [6]. The microbiota of seminal fluid has long remained poorly studied due to the low biomass of microorganisms and the difficulty of cultivating the microorganisms present by traditional methods [7,8]. Furthermore, classical microbiological analysis methods have been focusing exclusively on the identification of pathogenic microorganisms that are obviously causing infectious diseases [9].

Modern studies have revealed the presence of microbial communities in the testicular contents of clinically healthy individuals, which contradicts the traditional concept of sterility of these organs [10]. The sperm microbiome is a unique ecological niche where bacterial communities coexist with spermatozoa, influencing sperm quality and fertility [11,12,13,14,15,16]. For example, in humans, a link has been established between the sperm microbiome and abnormal sperm parameters. For instance, an increased abundance of *Lactobacillus iners* was observed in men with impaired sperm motility, indicating the potential role of specific microbial taxa in fertility [12].

The relevance of studying the bull semen microbiome using next-generation sequencing (NGS) is related to the need to move from searching for individual pathogens to analyzing the entire microbial ecosystem [7]. Microbiome analysis may detect dysbiosis that can lead to decreased fertility (e.g., through activation of inflammatory pathways), even if sperm quality is otherwise good [11].

Moreover, studying the link between microbial communities and standard sperm quality indicators may provide an opportunity to identify new causes of reduced fertilizing ability that were previously unknown.

The aim of the study was to determine the taxonomic composition and diversity in the sperm microbiome of sires and to establish its possible link with the main parameters of sperm quality.

## 2. Materials and Methods

### 2.1. Experimental Animals

The experiment was conducted on 21 Holstein bulls with varying reproductive quality, including subfertile individuals, in 2025 at the JSC “Nevskoe” stud farm (Russia, St. Petersburg). All bulls selected for the study were aged between 14 months and 2 years. The average live weight of the animals was 1100 kg. All bulls had no history of metabolic diseases (e.g., ketosis or acidosis), laminitis, inflammatory diseases of the reproductive system (balanoposthitis, vesiculitis, etc.), or other conditions that could affect the composition of their microbiota. A palpation examination was performed to confirm the absence of pathological changes in the scrotal organs and prostate gland. Bulls that had received antibiotics, hormonal drugs, probiotics, or other medications that could affect the microbiota composition within the last 3 months were excluded from the study.

The bulls were kept in individual stalls with access to automatic waterers and on straw bedding that was changed daily. Animals were fed twice a day. The diet of the experimental animals is presented in Table 1. The chemical composition of the diet is shown in Table 2.

Experimental animals were acclimatized to the study conditions for 15 days. During this time, the bulls’ general health, appetite, and behavior were carefully monitored to exclude any stress factors that could affect the microbiota composition. If signs of illness or deterioration in general condition were detected, the animal was excluded from the experiment.

Sperm microbiota sampling was performed twice weekly (in double cages) using an artificial vagina. Due to the primary objective of the study, namely, comparison of individual microbial profiles between animals, technical replicates for the same semen sample were not performed. Before sampling, the bulls’ preputial hair was clipped, the skin was treated with a sterile 0.9% NaCl solution, and the skin was dried with disposable sterile wipes. Sperm collection and processing procedures were performed under strict aseptic conditions using sterile instruments and disposable materials to minimize external contamination. IMV artificial vaginas (IMV Technologies LLC, L’Aigle, France) were sterilized. For sterilization, the disassembled artificial vagina was thoroughly washed to remove any remaining semen and lubricant using warm water and a neutral detergent and then rinsed thoroughly with deionized water. All components were then immersed in a container of water and brought to a boil. The boiling time was 30 min. After boiling, the components were carefully removed and dried on a clean, disinfected surface in a BAV-PCR-Laminar-S PCR cabinet with a UV air recirculator (Laminar Systems, Miass, Russia). The exposure time to ultraviolet rays was 30 min. All procedures were performed under aseptic conditions.

The lubricant used, Mediagel (Geltek-Medika LLC, Moscow, Russia), was tested for its antibacterial properties. Sterile filter paper discs (6 mm in diameter) were soaked in 20 µL of the lubricant and placed on the surface of nutrient agar with 1% peptone seeded with *Lactobacillus* spp., *Escherichia coli*, and *Staphylococcus* spp. (1 × 10^6^ CFU/mL). After 24 h of incubation at 37 °C, growth inhibition zones around the discs were measured. Sterile petroleum served as a control. Growth inhibition zones around discs containing the test lubricant were comparable to the inhibition zones of the test strains around discs containing the control petroleum (no significant differences, *p* > 0.05). Each component of the artificial vagina was thoroughly cleaned of any remaining lubricant or semen before sterilization. Samples were immediately placed in cryovials (Nunc, Thermo Fisher Scientific Inc., Waltham, MA, USA), frozen in liquid nitrogen (JSC Nevinnomyssk Azot, Nevinnomyssk, Russia), and transported to the BIOTROF+ laboratory (Russia, Leningrad Oblast, Tosnensky District) for storage at −80 °C until DNA extraction.

Our study was of a pilot nature, since obtaining ejaculate samples from genetically valuable sires with clinically confirmed and contrasting fertility status presents significant logistical and ethical challenges. In our study, semen samples from 21 bulls were analyzed. Previously, Cojkic et al. [17] conducted a similar study using a sample of 18 animals, which, nevertheless, demonstrated the ability to obtain representative data for identifying marker microbial clusters.

### 2.2. Sperm Analysis, DNA Extraction, and NGS

The obtained sperm samples were assessed according to standard quality parameters (volume, concentration, motility, morphology) immediately after receiving the sperm in the laboratory of JSC Nevskoe (St. Petersburg, Russia) using standard methods recommended for analyzing the reproductive function of farm animals [18]. Sperm concentration was determined using the SDM 6 photometer (Minitube GmbH, Tiefenbach, Germany). Morphological evaluation of spermatozoa was performed using light microscopy and Romanovsky-Giemsa staining. The percentages of normal spermatozoa and spermatozoa with head, neck, and tail abnormalities were calculated. Sperm motility was assessed visually under a Nikon H550S microscope (Nikon Corporation, Tokyo, Japan) and classified into four categories: fast progressive, slow progressive, non-progressive, and immotile according to WHO, 6th ed. [19]. Sperm DNA fragmentation was assessed by SCA using the halosperm G2 kit (Halotech DNA, S.L., Madrid, Spain). The fertilizing ability of the sperm was estimated based on the percentage of cows fertilized using a given batch of ejaculate.

To standardize the evaluation of bulls according to reproductive potential, the following criteria were used, based on reference values for bulls [20] (Table 3).

Total DNA was extracted from sperm samples using the Genomic DNA Purification Kit (Thermo Fisher Scientific, Inc.) following the manufacturer’s protocol. The DNA extraction process was conducted in a specialized clean room with HEPA air filters. Sterile consumables were used at all stages. Sterile swabs used for preputial swabbing were included as environmental control samples. Only sperm samples for which the negative controls were negative for microorganisms by NGS were allowed for further analysis. The microbial community composition was assessed using targeted NGS technology on the MiSeq platform (Illumina, Inc., San Diego, CA, USA) employing the following primers for the V3-V4 region of the 16S rRNA gene: forward primer, 5’-TCGTCGGCAGCGTCAGATGTGTATAAGAGACAGCCTACGGGNGGCWGCAG-5’; reverse primer, 5’-GTCTCGTGGGCTCGGAGATGTGTATAAGAGACAGGACTACHVGGGTATCTAATCC-3’. Metagenomic sequencing utilized the following reagents: Nextera^®^ XT Index Kit (Illumina Inc.) for library preparation, Agencourt AMPure XP PCR (Beckman Coulter Inc., Brea, CA, USA) for product purification, and MiSeq^®^ Reagent Kit v2 (500 cycles; Illumina Inc.) for sequencing. To assess the technical accuracy of the sequencing and library preparation process, one negative control (empty extraction buffer) and one positive control (a mixture of known bacterial strains) were included in each library batch. These control samples were analyzed in parallel with the test samples.

The NGS results (raw 16S rRNA reads) for this study have been deposited in the NCBI repository (Sequence Read Archive/BioProject) under the accession number PRJNA1416014 (https://www.ncbi.nlm.nih.gov/bioproject/PRJNA1416014, accessed on 16 April 2026, data type: Raw sequence reads (Multispecies), registration date: 29 January 2026) and into the SRA archive, submission number: SUB15974259 (https://account.ncbi.nlm.nih.gov/?back_url=https%3A//submit.ncbi.nlm.nih.gov/subs/sra/SUB15974259/overview, accessed on 16 April 2026).

### 2.3. Post NGS Computational Analysis

Following the import of raw sequences into the QIIME 2 environment (version 2025.4) (https://amplicon-docs.qiime2.org/en/stable/, accessed on 16 April 2026), paired-end reads were merged and subjected to quality control filtering using standard parameters. The Deblur algorithm (https://docs.qiime2.org/2024.10/plugins/available/deblur/index.html, accessed on 16 April 2026) was applied to eliminate sequence noise [21]. Denoising of raw reads was performed using the DADA2 algorithm. Sequences were trimmed to 250 bp for forward and reverse reads. No additional trimming was applied from the beginning of the reads. For phylogenetic reconstruction de novo, the MAFFT software suite (version 7) was employed, which included the alignment of masked reads [22,23]. Taxonomic classification of the microbial taxa was performed by referencing the SILVA 138 database [24,25]. Species-level classification based on the short 16S rRNA region is tentative and has limited reliability. The study’s main conclusions are based on taxonomy at the genus or family level. Estimates for α-diversity were calculated according to previously established protocols [26]. Various biodiversity metrics (observed features, Shannon, evenness, and Simpson) were determined using the integrated diversity-lib plugin (version 2024.10.0) within the QIIME 2 environment [27,28]. Visualization was performed in R using boxplots grouped by semen quality categories. Given the compositional nature of microbiome data, a centered log ratio (CLR) transformation with a pseudocount (0.001) was applied, followed by z-score normalization. Hierarchical clustering was performed using Euclidean distances and Ward’s minimum variance method (Ward.D2), and results were visualized as heatmaps with taxa as rows and samples as columns. Relative abundance was calculated as the percentage of the number of sequences of a given phylum to the total number of sequences in the sample.

Statistical evaluation and mathematical modeling of the data were conducted using RStudio (version 1.1.453) and Microsoft Excel XP/2003 [29]. These analyses incorporated multivariate analysis of variance (multi-factor ANOVA) to determine significance. Data are expressed as mean values (M) accompanied by the standard error of the mean (± SEM).

To assess differences in the overall microbiome composition between fertility groups, a Bray–Curtis distance matrix was calculated based on the relative abundance table of taxa. Sample ordination was performed using principal coordinate analysis (PCoA). The statistical significance of differences in microbial community composition between groups was assessed using permutational analysis of variance (PERMANOVA) with the adonis2 function of the vegan R package (version 2.7-3), with 999 permutations. To test the homogeneity of within-group variances, the betadisper test followed by a permutation test (PERMDISP) was used.

Analysis of differential taxa abundance was performed using the MaAsLin2 (Multivariate Association with Linear Models) package (version 1.12.0). Linear models with a fixed effect of sample quality were used to assess associations between the relative abundance of taxa and groups. To control for false positives, the Benjamini–Hochberg method was used for multiple comparison correction [30].

## 3. Results

Based on a comprehensive evaluation of ejaculate samples, including sperm concentration, morphology, motility, and fertilizing ability, each bull was assigned a quality category. Animals were divided into four conditional groups: unsatisfactory (I), satisfactory (II), good (III), and excellent (IV) sperm quality, characterized by varying fertility potential (Table 4). It is worth emphasizing that this classification was used as a tool for organizing data within this pilot study and does not claim to be universal.

NGS of the bovine sperm microbiome in the study generated a total of 157,012 16S rRNA reads (with a median of 2868 reads, min = 325, max = 25,453). After demultiplexing, the average number of reads per sample was 61,339 (median 50,097). After data preprocessing (quality filtering, denoising, and chimera removal), 157,012 reads, corresponding to 1902 amplicon sequence variants (ASVs), were used in the analysis. The average sequencing depth was 7477 reads per sample.

**Figure 1:**
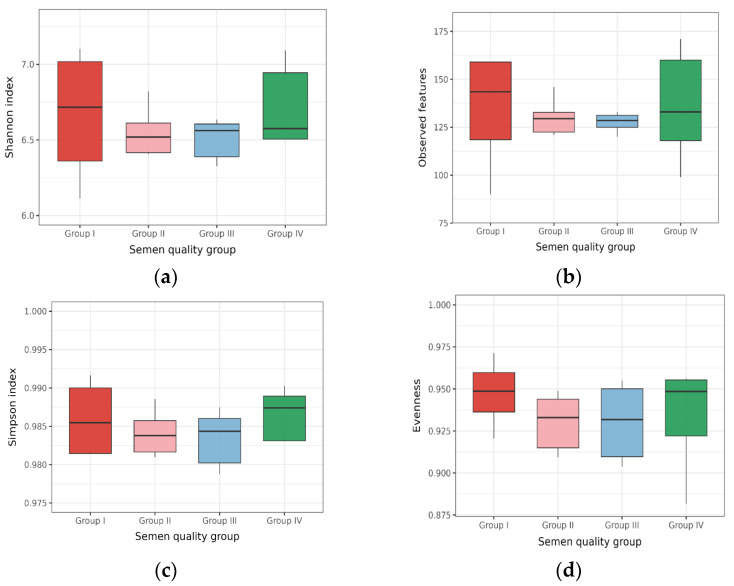
Values of the α-diversity index of the microbiome in the semen of Holstein sires (JSC Nevskoe, 2025) from different conditional groups; the groups: (**a**) unsatisfactory (I), (**b**) satisfactory (II), (**c**) good (III) and (**d**) excellent (IV) sperm quality. No statistically significant differences were observed between groups (*p* > 0.05).

The analysis showed that the composition of the sperm microbiome in bulls, including those with excellent fertility indicators, was quite diverse (Figure 1). The observed Shannon Diversity index values, ranging from 6.35 to 6.66, indicated significant species richness and uniformity of microorganism distribution in the semen of the bulls [31,32]. For example, the Shannon Diversity index values in our study were closer to the gut and oral microbiomes than to the skin and vagina samples [32]. This suggests that sperm are not only non-sterile in clinically healthy animals, as previously thought [33], but also show fairly great diversity in the bacterial community. The number of observed features also contributes to the assessment of the α-diversity characteristics of bull ejaculate samples [34]. In our study, the average number of observed features did not differ between groups. However, when analyzing this trait in individual bulls, it should be noted that its value was lowest in bull Parker_50224 from Group I (unsatisfactory ejaculate quality) at 90 and highest in bull Akhmat_3191 from Group IV (excellent ejaculate quality). Thus, individual factors may play a more important role in determining observed features in a particular animal than factors common to the entire group. The evenness and Simpson indices also showed no significant differences between groups.

**Figure 2:**
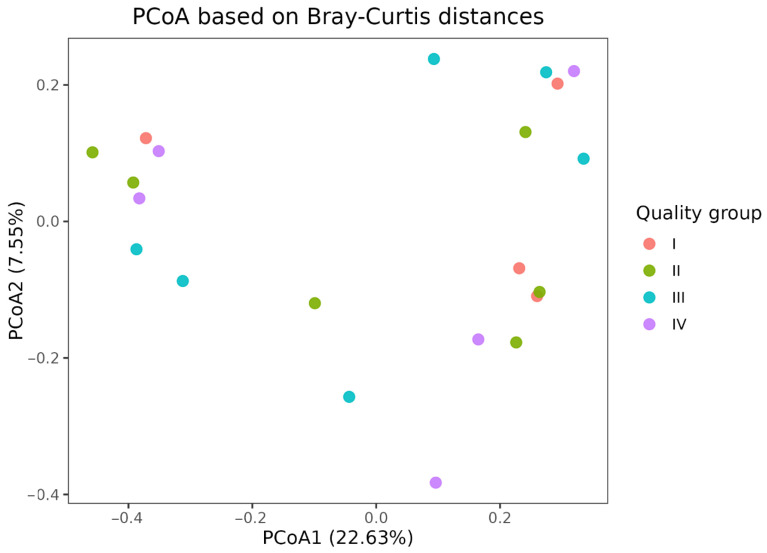
PCoA ordination of samples based on the Bray–Curtis distance calculated from the relative abundance of taxa. Dots represent individual samples; color denotes quality group (I–IV): unsatisfactory (I), satisfactory (II), good (III) and excellent (IV) sperm quality.

β-diversity analysis based on the Bray–Curtis distance revealed no statistically significant differences in microbiome composition between quality groups (PERMANOVA, R^2^ = 0.116, F = 0.745, *p* = 0.984) (Figure 2). Testing for homogeneity of within-group variances using the PERMDISP method also revealed no statistically significant differences between groups (F = 0.078, *p* = 0.969).

**Figure 3:**
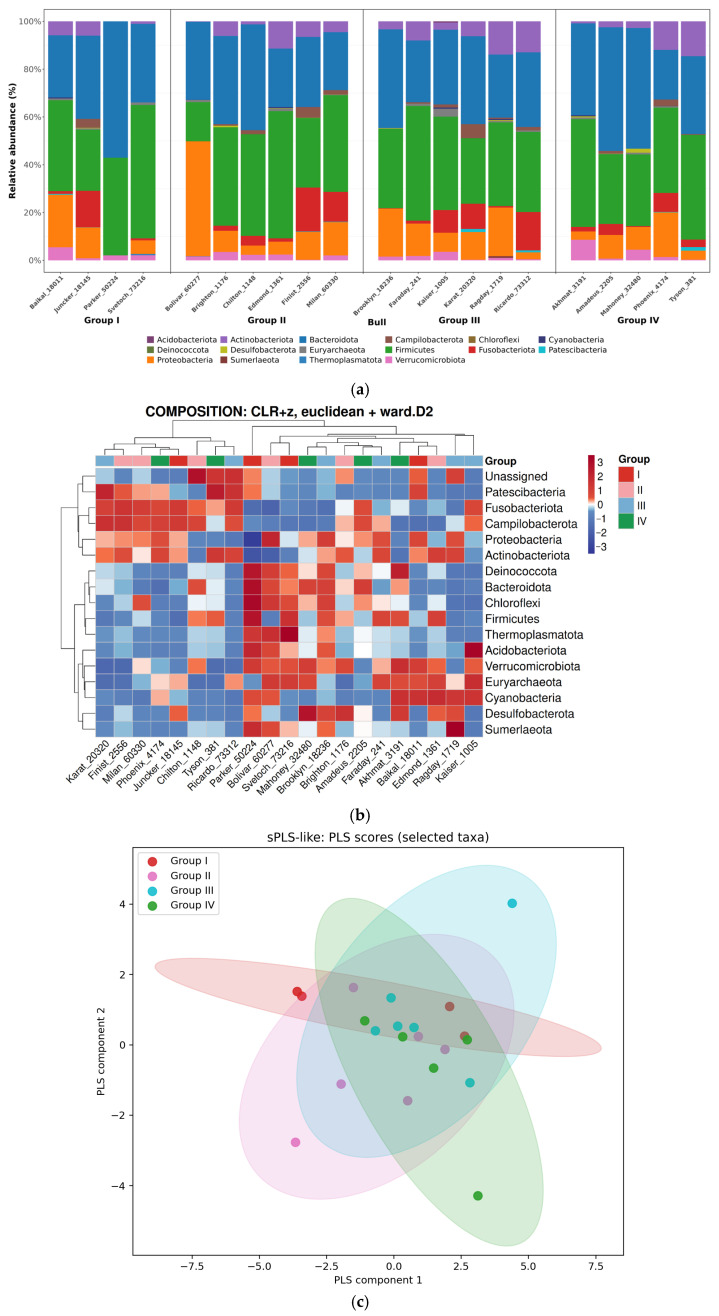
Content of bacterial phyla in the microbiome of the semen of Holstein sires (JSC Nevskoe, 2025) from different conditional groups. The groups: unsatisfactory (I), satisfactory (II), good (III) and excellent (IV) sperm quality: (**a**) Relative abundance of bacterial phyla depending on the experimental group; (**b**) Heatmap of the relative abundance of bacterial phyla, demonstrating the clustering of ejaculate samples based on their microbial composition. Color intensity corresponds to the z-score of the normalized relative abundance. On the color scale of relative abundance of microorganisms, red color corresponds to high relative abundance of the phylum, white color to abundance close to average, and blue color to low relative abundance of the phylum. Samples (horizontal axis) and taxa (vertical axis) are clustered using the Euclidean metric and the Ward D2 algorithm; (**c**) sPLS-like scores, reflecting the taxonomic composition of bacteria in the semen of bulls with different ejaculate qualities. Each dot represents a sperm sample, colored according to the ejaculate quality group. The coordinate axes show the sPLS (sparse Partial Least Squares) scores for the selected taxa, sPLS1 and sPLS2—the first and second components of sPLS, which explain the largest proportion of the variability in the bacterial composition data. Confidence ellipses provide a clear indication of whether groups are statistically distinct.

We identified at least 15 different bacterial phyla represented in the sperm samples (Figure 3a). The most common phyla represented in almost all samples included Bacteroidota, Firmicutes, Proteobacteria, Fusobacteriota, Actinobacteriota, and Verrucomicrobiota. It is worth noting that in all studied groups, the dominant phyla in terms of numbers were Bacteroidota and Firmicutes (Figure 3a). The relative content of Bacteroidota ranged from 20.8 ± 1.54% to 57.0 ± 3.94% and that of Firmicutes from 16.4 ± 1.80% to 55.8 ± 2.99%. Other, although less abundant, phyla were Proteobacteria (from 2.9 ± 0.35% to 48.1 ± 2.84%), Actinobacteriota (from absent to 14.5 ± 0.93%), and Fusobacteriota (from absent to 18.4 ± 1.36%). Previously, researchers have also shown that the diversity of bacteria in bull semen is high [35]. Thus, Jiang et al. [36] concluded that the most abundant phylum found in previous studies in rooster semen was Firmicutes, accounting for 62.73% of the relative abundance, followed by Actinobacteriota (25.17%) and Bacteroidota.

Some differences in phylum composition were identified between sire groups, as demonstrated by data visualization using sPLS-like scores analysis, which showed a tendency for samples to cluster according to relatedness to the ejaculate quality group (Figure 3c). It is noteworthy that the phyla Fusobacteriota and Actinobacteriota were completely absent only in the bull Parker_50224 from group I (unsatisfactory ejaculate quality) while being present in all other bulls studied. (Figure 3a,b), which may indicate dysbiotic disturbances in the sperm microbiome in this animal. This finding highlights the individual characteristics of each bull’s microbiome and suggests that, even within a group with identical ejaculate quality, the causes of decreased fertility may be different and related to the unique composition of the microbial community. However, overall, group IV (excellent ejaculate quality) had a higher relative abundance of Actinobacteriota (from 1.9 ± 0.12% to 14.1 ± 1.02%) compared to group I (*p* ≤ 0.05) (Figure 3b). This suggests that this bacterial phylum may have a positive effect on bull fertility. The identified association allows us to hypothesize a potential positive role for Actinobacteriota members in maintaining normal ejaculate biota. As a theoretical explanation, one can consider the fact that the immunomodulatory properties of some Actinobacteriota have been previously described in the scientific literature [37]. Immune balance in the reproductive tract is critical for protecting sperm from autoimmune attacks and inflammatory damage [38]. In addition, Actinobacteriota may produce various biologically active metabolites [39]. Hypothetically, these compounds may either directly improve sperm survival and functionality (e.g., antioxidants protect against oxidative stress) or indirectly influence fertility by maintaining an optimal environment in the reproductive tract [36]. Additionally, Actinobacteriota may promote competitive exclusion of potential pathogens or opportunistic microorganisms from seminal fluid by synthesizing a broad spectrum of antimicrobial compounds [40]. Thus, it can be assumed that the identified taxa could indirectly influence the ejaculate environment.

However, it is important to emphasize that our data do not provide information on functional activity, and other studies have also noted negative associations between the presence of Actinobacteriota members and sperm parameters [41]. This confirms the preliminary nature of our findings and the need for further species identification and functional analysis. In addition, members of this phylum are capable of producing isoprenoids, molecules that potentially cause fragmentation of sperm DNA [42].

**Figure 4:**
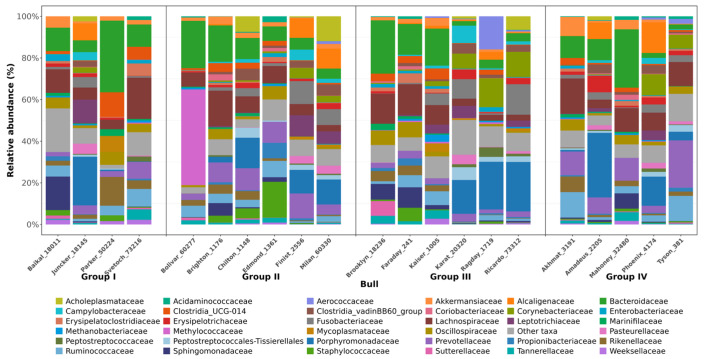
Relative abundance of bacterial families in the semen of Holstein sires (JSC Nevskoe, 2025); Groups: unsatisfactory (I), satisfactory (II), good (III) and excellent (IV) sperm quality.

These conflicting findings highlight the need to analyze the abundance of specific taxa within Actinobacteriota that are likely to exert positive or negative effects on bull fertility, as well as the mechanisms by which they show their effects. For example, three sires from Group IV, demonstrating characteristics of excellent ejaculate quality, were found to have a significant predominance of members of the *Corynebacteriaceae* family within the Actinobacteriota phylum (from 6.5 ± 0.52% to 10.9 ± 0.67%) (Figure 4). A similar trend was observed within Group III. While in Group I the average relative content of *Corynebacteriaceae* was only 0.93 ± 0.061%, in experimental Group II it was 1.6 ± 1.9%. Previous human studies have shown that members of this family are frequent inhabitants of the ejaculate of both healthy men and men with reproductive system diseases [43]. This suggests that *Corynebacteriaceae* may be part of the normal microbiota of the male reproductive tract [44]. Moreover, studies of *Corynebacterium* species inhabiting other biotopes reveal patterns of interactions with the host organism similar to those we identified. For example, representatives of this genus in the nasal cavity demonstrate interactions with both commensal and potentially pathogenic microorganisms [44]. In particular, some studies have noted a negative correlation between the presence of *Corynebacterium* and potentially pathogenic *Staphylococcus aureus* [45]. This indicates the potential ability of *Corynebacterium* to inhibit or eliminate unwanted microorganisms colonizing mucous membranes. Our results suggest that *Corynebacterium* may play an important role in maintaining microbial balance not only in the nasal cavity but possibly also in the reproductive tract, promoting its health and fertility.

**Figure 5:**
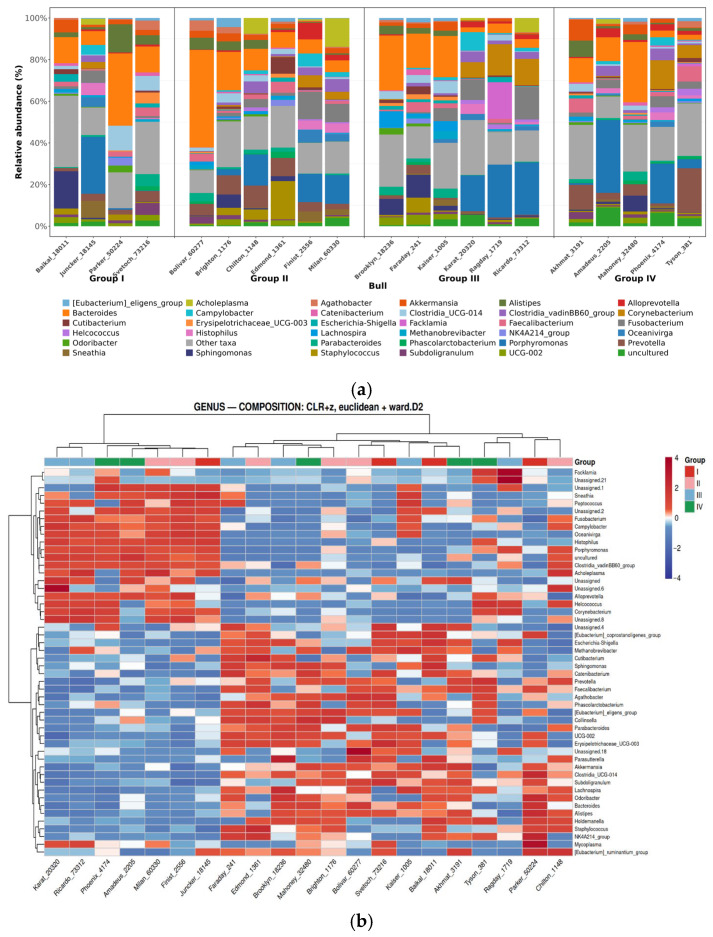
Relative abundance of bacterial genera in semen of Holstein sires (JSC Nevskoe, 2025); Groups: unsatisfactory (I), satisfactory (II), good (III) and excellent (IV) sperm quality; (**a**) Relative abundance of bacterial genera depending on the group. (**b**) Heatmap of the relative abundance of bacterial genera showing clustering of ejaculate samples based on their microbial composition. Color intensity corresponds to the z-score of the normalized relative abundance. On the color scale, red color corresponds to a high relative abundance of the genus, white color indicates abundance close to average, and blue color indicates low relative abundance of the genus. Samples (horizontal axis) and taxa (vertical axis) are clustered using the Euclidean metric and the Ward D2 algorithm.

At the genus level, genus *Faecalibacterium* was found to be frequently present in samples (Figure 5a,b). Although *Faecalibacterium* representatives were present in all studied samples, the average relative representation was slightly higher (1.5 times) in Group IV (2.1 ± 0.45%) compared to Group I samples (3.1 ± 0.52%) (Figure 4). Anti-inflammatory properties of some representatives of this genus, in particular *F. prausnitzii*, have been described previously in a study of the intestinal microbiome [46], suggesting that the species performs a similar function in the testes, reducing the risk of inflammatory processes that can negatively affect ejaculate quality [47]. *F. prausnitzii* produces butyrate, which is the main source of energy for colonocytes in the intestine and probably for sperm in the testes, and has an immunomodulatory effect [48].

The diversity of *Bacteroides* species found in bull semen, including *B. barnesiae*, *B. clarus*, *B. coprophilus*, *B. eggerthii*, *B. finegoldii*, *B. fragilis*, *B. kribbi*, *B. massiliensis*, *B. plebeius*, and *B. salyersiae*, may reflect their likely metabolic importance in the reproductive system. It is known that these bacteria are typical commensals of the animal intestine, where they participate in the fermentation of complex carbohydrates [49]. Hypothetically, the presence of this genus in semen could indicate its role in the metabolism of various compounds, which, in turn, may influence the availability of nutrients for sperm or other parameters of the environment.

**Figure 6:**
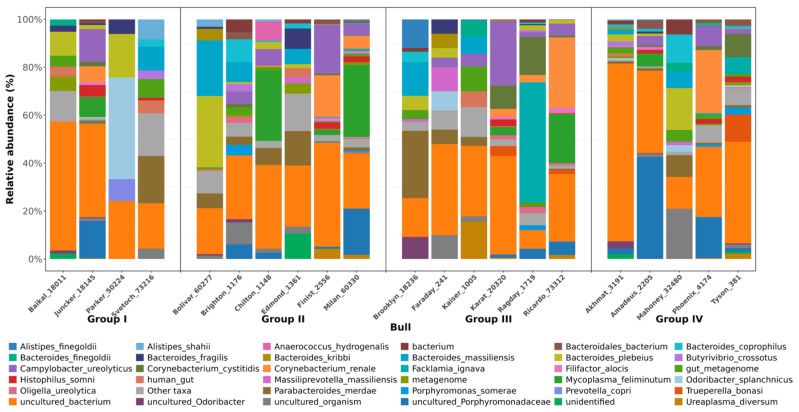
Relative abundance of bacterial species in semen of Holstein sires (JSC Nevskoe, 2025); Groups: unsatisfactory (I), satisfactory (II), good (III) and excellent (IV) sperm quality.

Relative abundance of bacterial species in semen of Holstein sires is shown on Figure 6. An interesting observation was the difference in the prevalence of the *B. fragilis* species between the conditional groups of the samples (*p* ≤ 0.05). In Group I, this species was detected in 75% of samples (3 bulls out of 4), while in Group IV, *B. fragilis* was detected in 20% of samples (1 bull out of 5). *B. fragilis* is considered a common intestinal commensal, but at the same time, some strains are opportunistic pathogens [50]. Certain strains of *B. fragilis* are associated with barrier function infections, particularly of the reproductive system [51]. Virulent strains of this species may carry genes encoding virulence factors such as *B. fragilis* enterotoxin, which causes inflammation, and capsular polysaccharide A, which can stimulate the production of anti-inflammatory IL-10 in the host [52].

Associations between taxon relative abundance and the study groups were assessed using MaAsLin2. After correction for multiple comparisons using the Benjamini–Hochberg procedure, no statistically significant associations were detected (q > 0.05).

However, analysis of uncorrected *p*-values suggested several consistent shifts in taxon abundance across the groups. Compared with group I, group II showed reduced relative abundance of the genera *Gardnerella* (*p* = 0.0023) and *Lactobacillus* (*p* = 0.0037). Group III was characterized by lower relative abundance of the genera *Alistipes* (*p* = 0.0028), *Roseburia* (*p* = 0.0035), and *Bacteroides* (*p* = 0.0051). In group IV, a decrease in the relative abundance of *Akkermansia* (*p* = 0.0012) and an increase in *Fusobacterium* (*p* = 0.0042) were observed.

These differences did not remain significant after multiple testing corrections and therefore should be interpreted as exploratory observations requiring confirmation in larger cohorts.

It has previously been shown that in Duroc boars, a decrease in ejaculate quality associated with changes in the ejaculate microbiota was observed, in particular with an increase in the number of another representative of the genus *Bacteroides* (*B. pyogenes*), as well as *Aerococcus*, *Gallicola*, *Ulvibacter*, and *Proteiniphilum* and with a decrease in the number of *Streptococcus gallolyticus* subsp. *macedonicus* [13]. On the contrary, earlier studies of the bacterial composition of bull semen showed that bulls with satisfactory spermograms had a higher number of sequences belonging to several genera, including *Bacteroides* [53]. The observed distribution of *B. fragilis* in our study may reflect a true trend or may be random. The increased frequency of *B. fragilis* in the group with unsatisfactory ejaculate parameters may suggest an association with subclinical reproductive tract disorders but does not constitute proof of its pathogenic role in this context.

Remarkably, *Akkermansia* spp. were detected in the semen of all sires, with a maximum of 8.5 ± 0.69% in bull Akhmat_3191 from Group I. Importantly, the highest average proportion among all groups was also found in Group I (*p* ≤ 0.05). Members of this genus are known for their ability to degrade mucin [54]. The presence of *A. muciniphila* is thought to influence ejaculate viscosity and sperm motility. Furthermore, *A. muciniphila* is known to modulate the immune system [55].

Individual sires were found to have increased levels of less common genera and species, which may indicate individual differences in their microbiota, which may, however, also be a factor influencing reproductive potential.

For example, the prevalence of *Collinsella* in samples differed between Group I and Group IV (*p* ≤ 0.05). In Group I, *Collinsella* spp. were detected in 50% of samples (2 out of 4). In Group IV, however, this genus was detected in 100% of samples. Human studies have previously shown that the presence of *Collinsella* has a beneficial effect on sperm motility, sperm count, and ejaculate viscosity [56]. On the other hand, some members of the genus, such as *C. aerofaciens*, are associated with inflammatory processes [57].

In addition, the breeding bull named Junker_18145 from Group I showed an increase (up to 1.29%) of *Histophilus somni* compared to other studied bulls (*p* < 0.05). This may indicate a subclinical infection and associated reduced fertility in the animal. Histophilosis is a common disease that causes septicemia in adult cattle and sudden death in calves [58]. The causative agent is *H. somni*, an opportunistic pathogen that can cause respiratory and reproductive diseases in cattle [59]. This microorganism was previously detected in the semen of beef bulls [60]. The virulent form of *H. somni* was isolated from the foreskin of a healthy bull and from the vagina of cows with clinical manifestations of granulosa vulvovaginitis and abortion [61]. This is particularly interesting because it has previously been shown that the microbiome of the male reproductive tract can not only influence sperm health but also be transmitted to the female during insemination, influencing the uterine microbiota and the likelihood of successful conception in ruminants [14,15,16].

## 4. Discussion

NGS of the semen microbiome of Holstein bulls revealed that the ejaculate of even clinically healthy animals without obvious fertility abnormalities is an ecological niche with a high level of biological diversity. This conclusion is supported by both the results of α-diversity analysis and taxonomic data identifying specific bacterial groups at various levels, from phyla to species. This refutes the concept of bacteria-free ejaculate in healthy animals and points to the existence of a complex microbial ecosystem whose role in reproductive function requires rethinking.

The microbial composition of bull sperm within a single farm demonstrated a level of similarity but at the same time was characterized by differences within groups with different levels of fertility (ejaculate quality and fertilizing ability). The study identified putative key taxa associated with ejaculate quality. Thus, the increased levels of Actinobacteriota in the group with excellent ejaculate quality suggest a potential positive effect on fertility. Given the known anti-inflammatory properties of some *Faecalibacterium* representatives, their presence may be considered as a potential factor in reducing inflammation in the testes. This allows us to classify this genus as a likely representative of the normal semen biota. The data obtained may also indicate a potential role for *Collinsella* spp. in maintaining microbial balance and promoting fertility. The diversity of *Bacteroides* was also revealed, which may reflect their likely metabolic significance. Given that *B. fragilis* may be represented by opportunistic strains, its high prevalence in the group with poor semen quality may indicate dysbiosis. However, due to the relatively small number of animals studied (*n* = 21), the identified distribution of these taxa may not reflect the true trend and may be random. Therefore, further research is necessary. Also, the microbiome changes may be the primary cause of fertility decrease or a secondary effect of subclinical inflammatory processes.

It is also worth noting that all animals were obtained from a single farm and maintained under identical conditions. Although this allowed for control of environmental variables, the results may reflect farm-specific effects on the semen microbiome. Further research is needed to determine whether these microbial patterns are universal across different geographic regions and management systems.

Our results also revealed individual differences in the microbial composition of individual animals, likely due to factors such as the state of the immune system, the presence of subclinical infections, etc. Thus, an increase in the number of *H. somni* in a bull from the group with unsatisfactory ejaculate quality allows us to consider this as an indicator of a latent infection, potentially reducing fertility. Our study was conducted on a relatively small cohort of 21 bulls. Although this number was sufficient to identify significant trends and form four conditional groups of ejaculate quality, a larger sample size is needed to further confirm the statistical reliability of the observed correlations, particularly for rare taxonomic groups and individual variations; that is, it is important to establish whether this observation is random or whether there is a statistically significant relationship between the increase in the content of individual bacterial taxa and changes in the quality of ejaculate.

Further research should focus on clarifying the spectrum of marker taxa and studying their functional potential to determine the precise mechanisms of their influence on sperm physiology.

## 5. Conclusions

The data obtained from the study on taxonomic markers associated with high and low fertility rates is consistent with the possibility of conducting routine monitoring of the semen microbiome. Regular NGS analysis would allow the detection of potential deviations in the semen microbiome and could become a valuable additional preventive tool. The risk of semen contamination at various stages (collection, dilution, and cryopreservation) cannot be completely eliminated, even taking into account the strictness of the protocols. Microbiome monitoring may serve as a highly sensitive indicator of the effectiveness of these procedures. Similarly, tracking the dynamics of pathogenic taxa after antimicrobial or probiotic therapy in a breeding bull can provide an objective picture of the success of treatment and prevention, complementing bacterial culture.

## Figures and Tables

**Table 1:** Feed ingredients of bulls’ diets.

Raw Material	Mass (kg)	Percentage (%)
Mixed-grass hay	11.00	58.698
Compound feed for bulls (JSC Leningradsky Bread Products Plant, St. Petersburg, Russia)	5.000	28.881
Chicken eggs	0.240	1.281
Premix for bulls (JSC Nevskoe, St. Petersburg, Russia)	0.100	0.534
Sugar	0.100	0.534
Dry whey powder	0.300	1.601
Carrot	2.000	10.672
Total	18.740	100.00

**Table 2:** Chemical composition of bulls’ diets.

Nutrient	Minimum Value	Actual Content
Dry matter (g/kg)	13,400.00	15,506.44
Protein (g/kg)	2726.00	2478.60
Digestible protein (g/kg)	1653.00	1736.02
Rumen degradable protein (g/kg)	1201.00	1963.23
Rumen Undegradable Protein (g/kg)	1526.00	479.97
Fat (g/kg)	537.00	285.81
Fiber (g/kg)	2674.00	4111.98
Ash (g/kg)	-	1089.51
Sugar (g/kg)	1649.00	990.61
Starch (g/kg)	1814.00	1312.65
Starch + sugar (g/kg)	-	2303.25
Metabolic energy, MJ/kg	134.00	121.11
Calcium (g/kg)	80.00	106.02
Phosphorus (g/kg)	70.00	74.75
Sodium (g/kg)	-	29.13
Magnesium (g/kg)	44.00	35.35
Salt (g/kg)	80.000	49.750
Sulfur (g/kg)	55.00	26.54
Vitamin A (IU/kg)	-	25,000
Vitamin D3 (IU/kg)	16,500	8126
Vitamin E (mg/kg)	400	535.38
Iron (mg/kg)	735.00	2031.89
Manganese (mg/kg)	669.00	997.04
Zinc (mg/kg)	536.00	551.99
Copper (mg/kg)	128.00	180.61
Iodine (mg/kg)	10.00	23.53
Selenium (mg/kg)	-	2.19
Cobalt (mg/kg)	10.00	14.47
Volume (kg)	-	19

**Table 3:** Criteria for assessing the sperm quality and the fertilizing ability of bulls.

Quality Category	Sperm Concentration (Billion/mL)	Morphology (Normal Forms, %)
Very Low	-	<45
Low	<0.7	45–55
Below Average	0.7–0.9	55–60
Average	0.9–1.1	-
Satisfactory	-	60–67
Good	1.1–1.3	67–75
Excellent	>1.3	>75
Above excellent	-	-

**Table 4:** Distribution of the studied bulls into conditional groups in accordance with internal criteria of ejaculate quality within the framework of the study.

Group Number	Group Description	Criteria	List of Bulls
I	Unsatisfactory ejaculate quality	At least one of the indicators is significantly below the threshold values, indicating serious fertility problems	Junker_18145, Parker_50224, Baikal_18011, Svetoch_73216
II	Satisfactory ejaculate quality	Most (or all) indicators are within acceptable limits or slightly below optimal values.	Brighton_1176, Edmond_1361, Bolivar_60277, Finist_2556, Chilton_1148, Milan_60330
III	Good ejaculate quality	Most indicators are good or excellent, while others may be average or slightly below average.	Brooklyn_18236, Faraday_241, Ricardo_73312, Karat_20320, Ragday_1719, Kaiser_1005
IV	Excellent ejaculate quality	All indicators (morphology, motility, concentration, fertility) are high (>75% or in the upper limits), indicating excellent fertility properties	Amadeus_2205, Mahoney_32480, Akhmat_3191, Phoenix_4174, Tyson_381

## Data Availability

The NGS results (raw 16S rRNA reads) for this study have been deposited in NCBI repository (Sequence Read Archive/BioProject) under the accession number: PRJNA1416014 (https://www.ncbi.nlm.nih.gov/bioproject/PRJNA1416014, accessed on 16 April 2026, data type: Raw sequence reads (Multispecies), registration date: 29 January 2026), and into the SRA archive, submission number: SUB15974259 (https://account.ncbi.nlm.nih.gov/?back_url=https%3A//submit.ncbi.nlm.nih.gov/subs/sra/SUB15974259/overview, accessed on 16 April 2026).

## Abbreviations

The following abbreviations are used in this manuscript:

NGS	Next-Generation Sequencing
DNA	Deoxyribonucleic Acid
RNA	Ribonucleic Acid
NCBI	National Center for Biotechnology Information
M	Mean Values
SEM	Standard Error of the Mean
CLR	Centered Log Ratio
IL-10	Interleukin-10
